# Patterns of Vulnerability: Frailty, Multimorbidity, and Physical Health-Related Quality of Life in Institutionalised Older Adults

**DOI:** 10.3390/healthcare14111491

**Published:** 2026-05-27

**Authors:** Noelia Durán-Gómez, Miguel Ángel Martín-Parrilla, Jesús Montanero-Fernández, Casimiro Fermín López-Jurado, Lydia Rodríguez-Rivero, Macarena C. Cáceres

**Affiliations:** 1 Departamento de Enfermería, Facultad de Medicina y Ciencias de la Salud, Universidad de Extremadura, 06005 Badajoz, Spain; nduran@unex.es (N.D.-G.); casimirolj@unex.es (C.F.L.-J.); mcaceres@unex.es (M.C.C.); 2 Grupo de Investigación Traslacional Biomédica y Sociosanitaria (CTS064), 06005 Badajoz, Spain; 3 Instituto Universitario de Investigación Biosanitario de Extremadura (INUBE), 06005 Badajoz, Spain; 4 Departamento de Enfermería, Centro Universitario de Plasencia, Universidad de Extremadura, 10600 Plasencia, Spain; 5 Departamento de Matemáticas, Facultad de Medicina y Ciencias de la Salud, Universidad de Extremadura, 06005 Badajoz, Spain; jmf@unex.es; 6 Servicio Extremeño de Salud, 06800 Mérida, Spain; lydia.rodriguez@salud-juntaex.es

**Keywords:** aged, 80 and over, frail elderly, multimorbidity, quality of life, homes for the aged, activities of daily living, geriatric nursing

## Abstract

**Highlights:**

**What are the main findings?**

- Frailty, multimorbidity, and advanced age were associated with poorer physical health-related quality of life in institutionalised older adults.
- Distinct vulnerability patterns were observed according to age, frailty status, and multimorbidity burden within the Physical Health domain.

**What are the implications of the main findings?**

- Integrating frailty and multimorbidity assessment into comprehensive geriatric evaluation may improve the identification of vulnerable residents in long-term care facilities.
- Early identification of frailty-related vulnerability may help guide targeted interventions to maintain physical health and quality of life in institutionalised older adults.

**Abstract:**

Background/Objectives: Population ageing is accompanied by an increasing burden of multimorbidity and frailty, both of which are consistently associated with poorer health-related quality of life (QoL). Although several geriatric domains influence QoL in older adults, their combined association remain insufficiently explored in institutionalised populations. This study aimed to examine the independent and combined associations between age, multimorbidity, frailty, and QoL in institutionalised older adults and to explore which quality-of-life domain was most strongly associated with geriatric assessment variables. Methods: A cross-sectional study was conducted in 72 institutionalised older adults in Spain. Multimorbidity (number of chronic conditions), frailty (Fried phenotype), functional status, nutritional status, fall risk, and QoL were assessed using validated instruments, including the World Health Organization Quality of Life questionnaire. Pearson correlations and canonical correlation analysis were used to examine relationships between geriatric assessment variables and QoL domains. Analysis of variance and regression tree were subsequently applied to explore associations affecting the Physical Health domain. Results: A correlation analysis identified the Physical Health domain as the QoL dimension most strongly associated with geriatric variables. On the other hand, frailty, age and number of chronic diseases turned out to be the most explanatory in our study and were classified: the first according to the standard protocol, and the other two using a regression tree. Then, a three-way additive ANOVA explained 36.4% of the variance, with age as main influential. Namely, we estimate that the poorest QoL occurs in subjects over 84 who have more than three chronic conditions and are classified as frail. However, this is not a validated clinical decision rule since these cutoff points may vary in other samples. Conclusions: In this sample of institutionalised older adults, age emerged as the main variable associated with lower physical QoL, multimorbidity contributes to the cumulative burden of disease, and frailty may reflect the systemic decline in physiological reserves.

## 1. Introduction

Following the post-pandemic recovery, global life expectancy reached 73.3 years in 2024 and is projected to rise to 77.4 years by 2054 [1], within a broader context of accelerated population ageing that is particularly evident in Europe. Adults aged 65 years and older represent the fastest-growing demographic group worldwide and are expected to outnumber those under 18 in the second half of the century [1]. In the European Union, the proportion of people aged 65 years and older has increased from approximately 16% in 2004 to over 21% in 2024 and is projected to approach 30% by mid-century [2]. A similar demographic shift is observed among those aged 80 years and over, whose numbers are projected to increase substantially in the coming decades both globally and across Europe, further intensifying demands on health and long-term care systems [1,2].

The global rise in life expectancy has been accompanied by population ageing and an increasing burden of chronic conditions. With advancing age, the accumulation of chronic diseases leads to increasing multimorbidity and to more frequent functional decline. Evidence shows that multimorbidity is strongly associated with a higher prevalence of frailty among older adults, reflecting the interaction between biological aging and chronic disease burden [3,4]. As populations age, these intertwined processes are expected to increase the number of older individuals living with frailty. In this context, age not only is associated with a chronological marker but also as a determinant that amplifies the cumulative burden of disease and vulnerability, shaping the clinical expression of both multimorbidity and frailty. Multimorbidity, commonly defined as “the coexistence of two or more chronic conditions, where each must be a non-communicable disease, a mental health disorder, or an infectious disease of long duration” [5], has emerged as a major public health challenge in ageing populations. Recent evidence indicates that the global prevalence of multimorbidity among individuals aged 60 years and older is approximately 51% (95% CI: 44.1–58.0), highlighting the substantial burden of coexisting chronic conditions in this age group [6].

Although there is no universally accepted definition of frailty, the ADVANTAGE Joint Action systematic review describes it as an age-associated state of increased vulnerability to stressors. This condition results from cumulative declines across multiple physiological systems and leading to a higher risk of adverse outcomes such as disability, hospitalization, institutionalization, and death. Frailty is widely recognised as a multidimensional geriatric syndrome and an emerging public health priority in Europe in the context of demographic ageing [7]. Global frailty prevalence has been shown to reach 18% across the European countries. Rates are substantially higher among institutionalised older adults [8], where prevalence frequently exceeds 60%. These higher rates reflect both the greater clinical complexity of institutionalised populations and methodological differences across studies. Prevalence estimates vary according to the assessment tool used, with physical phenotype measures generally producing lower rates than multidimensional or deficit accumulation approaches, which limits comparability between studies [7].

Beyond its role as a marker of vulnerability, frailty has been consistently associated with advancing age and with the presence of comorbidity or multimorbidity [9], suggesting a complex and bidirectional relationship in which chronic disease burden and biological ageing reinforce each other. Importantly, this interaction has significant implications for health-related quality of life (QoL), as both multimorbidity and frailty have been shown to negatively impact physical, psychological, and social well-being [10,11]. Recent longitudinal evidence indicates that multimorbidity is associated with poorer QoL, partly through its impact on functional limitations [12], while population-based analyses further show that specific multimorbidity patterns are linked to worse QoL in older adults [13]. Thus, understanding the interrelationship between age, multimorbidity, frailty, and QoL is essential for addressing the broader consequences of population ageing and for guiding person-centred health and social care strategies.

QoL is a multidimensional construct encompassing individuals’ perceptions of their physical, psychological, and social functioning, reflecting both positive and negative aspects of health and well-being [14]. Beyond serving as an indicator of disease burden, QoL has become a central outcome in geriatric research, as contemporary healthy ageing frameworks emphasize functional ability, autonomy, and participation as core components of well-being in later life [15]. Its assessment provides valuable information for identifying population needs and informing clinical decision-making, resource allocation, and health policy. In institutionalised older adults, QoL may be further influenced by environmental context, care models, and levels of dependency, making it a particularly sensitive marker of overall health status. In the present study, QoL was operationalized using the WHOQOL-BREF, a multidimensional instrument that captures physical, psychological, social, and environmental domains of perceived QoL.

In addition to multimorbidity and frailty, other geriatric domains substantially influence QoL in older adults. Functional status, assessed through basic and instrumental activities of daily living (BADL and IADL), is a key determinant of perceived well-being, as limitations in everyday functioning are independently associated with lower QoL in European older populations [12,16]. Falls and impairments in balance and coordination, and gait performance—highly prevalent among frail and multimorbid individuals—further contribute to functional decline and reduced QoL, with robust evidence linking fall risk and gait–balance performance to poorer physical and psychosocial outcomes [17]. Moreover, nutritional risk, commonly assessed using the Mini Nutritional Assessment (MNA), has been independently associated with lower QoL and greater vulnerability in older adults [18]. These interrelated domains frequently coexist in long-term care settings, compounding clinical complexity and potentially amplifying the negative impact of multimorbidity and frailty on overall QoL.

Although a growing body of evidence demonstrates that both multimorbidity and frailty negatively affect QoL in older adults, most available studies have focused on community-dwelling populations. Institutionalised older individuals constitute a particularly vulnerable subgroup, characterized by greater clinical complexity, functional dependence, cognitive impairment, and distinct environmental and care-related conditions that may modify the relationship between health status and perceived QoL. To date, limited research has examined the independent and combined contributions of multimorbidity and frailty to QoL in long-term care settings, particularly within a multivariate and predictive framework.

Given the central role of nursing professionals in the comprehensive assessment and management of frailty, multimorbidity, functional decline, nutritional risk, and fall risk in these contexts, clarifying these relationships is essential to inform targeted, person-centred care strategies. Therefore, the aim of this study was to examine the independent and combined associations between multimorbidity, frailty, and QoL among institutionalised older adults, while accounting for additional interrelated geriatric domains that may influence perceived QoL. Additionally, it sought to explore potential patterns of vulnerability within a hypothesis-generating framework.

## 2. Materials and Methods

### 2.1. Design

An observational cross-sectional study was conducted in residential and long-term care facilities for older adults in Spain between 2023 and 2024. The study was reported in accordance with the STROBE [19] reporting guidelines.

### 2.2. Setting, Sampling, and Eligibility Criteria

The initial sample comprised 81 individuals; however, 9 did not complete the assessment and were excluded from the analysis, resulting in a final analytical sample (*N* = 72). Recruitment was conducted using a non-probability convenience sampling strategy. All participants who completed the study met the established eligibility criteria and provided informed consent prior to participation.

Eligibility criteria required participants to be 65 years of age or older, reside in the selected institutions, and be able to participate in the study procedures. Additionally, a minimum institutionalization period of six months was required to ensure residential stability. Exclusion criteria included severe cognitive impairment or physical limitations that prevented reliable assessment, acute or terminal medical conditions, and documented non-adherence to medical treatments or institutional regulations, as persistent non-adherence could compromise clinical stability and affect the reliability of functional and QoL assessments.

### 2.3. Data Collection

Data were collected in person at the participating residential and long-term care facilities by a single trained researcher who was unaware of the study hypotheses to minimize potential information bias. The investigator, a registered nurse with postgraduate education in health sciences, had received formal training in conducting clinical interviews, performing anthropometric measurements, and administering standardized questionnaires.

To limit recall bias, questionnaires were completed immediately following each structured interview, according to standardized written and verbal instructions. All procedures were conducted in private, quiet rooms within each facility to preserve confidentiality and minimize social desirability bias. The researcher supervised the entire process to ensure consistency across participants.

### 2.4. Instruments and Measures

A structured, study-specific sociodemographic questionnaire was designed to collect essential participant information, including age, sex, educational attainment, marital status, occupational status, and lifestyle-related variables. The instrument was tailored to the study objectives to ensure relevance and comprehensive data collection. Participants provided responses directly, with assistance offered when required to facilitate accuracy.

Clinical history data were obtained through structured interviews complemented by a review of medical records. Key variables included chronic conditions—such as hypertension, diabetes mellitus, dyslipidaemia, and cardiovascular disease—current pharmacological treatments, and previous hospital admissions. Multimorbidity was operationally defined as the number of chronic conditions recorded for each participant [20]. Information was corroborated against clinical documentation to improve reliability. These data were used to describe participants’ health profiles and to adjust for potential confounding variables in subsequent analyses.

Validated Spanish versions of standardized instruments were subsequently administered to assess frailty, fall risk, nutritional status, functional independence, balance and gait performance, and QoL.

To measure frailty, the Fried frailty phenotype was used. This operational definition conceptualizes frailty as a biological syndrome of decreased physiological reserve and resistance to stressors. It evaluates five criteria: unintentional weight loss, self-reported exhaustion, low physical activity, muscle weakness (assessed through handgrip strength using a calibrated dynamometer), and slow gait speed (measured over a 6 m walking distance according to standardized protocols, with cut-off values adjusted for sex and height). Participants meeting three or more criteria were classified as frail; those with one or two criteria as pre-frail; and those with none as robust. Spanish adaptations of the Fried criteria have demonstrated adequate construct and predictive validity for adverse outcomes such as disability, institutionalization, hospitalization, and mortality in older Spanish cohorts, as well as satisfactory inter-rater reliability when standardized measurement procedures are applied [21].

To assess fall risk, the Downton Fall Risk Index was administered. This instrument evaluates multiple intrinsic and extrinsic risk factors, including previous falls, medication use (e.g., sedatives, antihypertensives, diuretics), sensory deficits (visual and auditory impairments), cognitive status, and gait stability. Scores are summed to obtain a total risk score (range 0–11), with higher scores indicating greater risk of falling. A cut-off score of ≥3 was used to identify high fall risk. The Spanish version has shown acceptable internal consistency and adequate predictive validity in institutionalised older adults, with suitable sensitivity and specificity for identifying individuals at increased risk [22].

Nutritional status was evaluated using the full version (0–30 points) of the MNA. This instrument includes anthropometric indicators (body mass index, weight loss, arm and calf circumference), dietary assessment, global clinical evaluation (mobility, medication use, acute illness or stress), and subjective self-perception of health and nutrition. Based on the total score, participants are categorized as well-nourished (24–30), at risk of malnutrition (17–23.5), or malnourished (<17). The Spanish validation of the MNA has demonstrated high sensitivity and specificity for detecting malnutrition and risk of malnutrition in both community-dwelling and institutionalised older adults, as well as strong concurrent validity with clinical and biochemical nutritional parameters [23].

Independence in instrumental activities of daily living (IADL) was assessed using the Lawton–Brody Scale (range 0–8, higher scores indicating greater independence). This scale assesses the ability to perform complex daily tasks required for autonomous community living, including telephone use, shopping, meal preparation, housekeeping, laundry, transportation, medication management, and financial management. Higher scores indicate greater functional independence. The Spanish-adapted version has demonstrated good internal consistency, interrater reliability, and construct validity in geriatric populations, supporting its use in residential care settings [24].

Functional independence in basic activities of daily living (BADL) was assessed using the Barthel Index (range 0–100, with higher scores reflecting greater independence). This instrument evaluates ten fundamental domains: feeding, bathing, grooming, dressing, bowel control, bladder control, toilet use, transfers, mobility, and stair climbing. The total score reflects the degree of dependence, ranging from total dependence to complete independence. The Spanish version of the Barthel Index has shown high in-ter-observer reliability and robust criterion validity in older populations and is widely used in clinical and research contexts in Spain [25].

Balance and gait performance were evaluated using the Tinetti Performance-Oriented Mobility Assessment (POMA) (range 0–28), comprising a balance subscale (maximum 16 points) and a gait subscale (maximum 12 points). Lower total scores indicate poorer performance and greater fall risk. For descriptive purposes, scores were categorized as no fall risk (≥25), low fall risk (19–24), and high fall risk (<19). The Spanish adaptation has demonstrated good inter-rater reliability and predictive validity for falls in institutionalised older adults [26].

Perceived health-related QoL was assessed using the WHOQOL-BREF questionnaire. This 26-item instrument evaluates four domains—physical health, psychological health, social relationships, and environment—along with two general items on overall QoL and general health. Each item is rated on a five-point Likert scale, and domain scores were calculated and transformed to a 0–100 scale according to WHO scoring guidelines, with higher scores indicating better perceived QoL across domains. The Spanish version has demonstrated adequate internal consistency, construct validity, and cross-cultural equivalence in older Spanish populations [27].

### 2.5. Statistical Analysis

The data was analysed using SPSS version 31 and R version 4.5.1. The sample size (*N* = 72) was not based on a prior calculation, but rather as many subjects as possible were analysed. In any case, this size allows for the detection of a minimum correlation of ±0.375 with a significance level of α = 0.05 and a power of 1 − β = 0.90. After a descriptive analysis of all variables, a simple correlation analysis was performed between the most important outcomes. A Bonferroni Post Hoc correction was applied to avoid type I errors. This correction implied a severe reduction that was consistent with a canonical correlation analysis.

The main objective of this study was to find a parsimonious model that explains quality of life in elderly subjects based on some of the most popular geriatric scales (mentioned above), considering some possible confounding variables, for example age, gender, chronic conditions and others. To avoid overfitting in the process, we chose to simplify the model as much as possible using stepwise selection algorithm and regression tree (CART) with a minimum of 10 subjects per filial node and 20 per parental node. Once the most relevant variables had been selected, an ordinary least squares (OLS) linear model was applied in its various forms (multiple regression, ANCOVA, and multifactorial ANOVA) to identify the model that best fit the data.

## 3. Results

Table 1 presents the descriptive statistics for all variables in the study. Numerical variables were expressed as mean ± standard deviation and median (IQR) (when showed a strong asymmetry). The categorical variables were expressed as frequency and percentage.

The objective of this study is to identify the most significant relationships between quality of life and certain variables that are typically of interest to older adults. In this case, there are 23 variables involved in a sample of *N* = 72 subjects. To avoid excessive overfitting and multiple Type I errors, a drastic reduction was applied, which was verified using alternative multivariate methods, as will be explained below.

The results of the correlation analysis between the main variables, expressed as Pearson’s r, are presented in Table 2. In this table, the variables are divided into two groups: explanatory variables (age, frailty, fall risk, nutritional status, IADL, BADL, balance and gait) and the four domains of QoL. Correlations among the geriatric scales, on the one hand, and among the QoL domains, on the other, could be considered expected. The primary objective was to examine the 28 cross-correlations between both groups, highlighted in the rectangle. Although many of these correlations were statistically significant, the application of a Bonferroni correction led us to focus just on those with a *p*-value lower than 0.0018, corresponding to an absolute Pearson’s r greater than 0.350 (highlighted in bold in Table 2). Under this criterion, negative correlations were observed between the Physical Health QoL domain and age, frailty, and fall risk, whereas direct correlations were found between the Physical Health QoL domain and BADL as well as balance and gait.

According to these results, the remaining correlations should be excluded from the rest of the analysis. To confirm this point of view, a canonical correlation analysis was performed. This yielded a single significant canonical correlation (r = 0.609, *p* = 0.018), indicating that the relationship between both groups of variables could be adequately summarized as a simple correlation between weighted means of the respective groups. Specifically, the weights considered in this calculation were, on the one hand, −0.762 for Age, −0.802 for Frailty scale, −0.649 for Downton scale, 0.719 for BADL scale, and 0.699 for Tinetti scale, compared to only 0.289 for MNA scale and 0.218 for Lawton–Brody scale. This result suggested that the latter two variables should be removed from the rest of the analysis. On the other hand, the weight for the Physical Health QoL domain was 0.932, compared with 0.512 for the Psychological Health QoL domain, 0.491 for the Social Relationships QoL domain, and 0.487 for the Environment QoL domain. This does not imply that the Psychological Health QoL domain, the Social Relationships QoL domain, and the Environment QoL domain are not correlated with the geriatric scales (as shown in Table 2), but their correlations are clearly lower than those corresponding to the Physical Health QoL domain. Due to the modest sample size, it remains uncertain whether these associations reflect a common underlying relationship with QoL or potential type I error.

Therefore, since the canonical correlation analysis confirmed the reduction following the Bonferroni correction, the OLS analysis presented below will only consider a predictive model for the Physical Health Quality of Life domain based on age, frailty, risk of falls, BADL and balance and gait.

First, due to the presence of obvious collinearity issues, a stepwise variable selection process was performed in a multiple linear regression model. As a result, frailty (*p* = 0.002) and age (*p* = 0.004) remained in the model, with an R^2^ of 0.299, while fall risk score (*p* = 0.804), BADL (*p* = 0.526), and balance and gait (*p* = 0.521) were excluded. This does not mean that these variables were unrelated to the domain of health-related quality of life; rather, their partial contribution to explaining that domain—beyond what is jointly explained by age and frailty—was not sufficiently significant in this sample.

Second, the rest of the variables listed in Table 1 were sequentially introduced into the model (ANCOVA) to assess potential confounding effects and improve model fit. Among these, only the number of chronic conditions was statistically significant (*p* = 0.022) when categorized into two groups (up to three vs. more than three). Frailty and age remained in the model. With the inclusion of chronic conditions, the coefficient of determination R^2^ increased to 0.350.

However, residual analysis revealed a poor fit with the assumptions of the OLS model, primarily due to non-linearity and skewness in the distribution of the frailty score. To address these issues and improve model adequacy, age and frailty were subsequently analysed as categorical variables. Categorization was also adopted to improve interpretability and model stability within the context of the exploratory analysis. Frailty was categorized into non-frail, pre-frail, and frail, as previously defined; age was dichotomized into ≤84 years and >84 years and number of chronic conditions into ≤3 and >3, based on a regression tree analysis (see Figure S1 in Supplementary Materials). It was carried out with a minimum of 10 subjects per final node, using the Physical Health QoL domain as the dependent variable and age and number of chronic conditions (both treated as continuous variables), together with categorized frailty status, as predictors. However, as noted in Section 4.7, these cutoff points should be valued with caution since they were not derived from a large sample. In fact, as a “bagging” validation method, the technic was applied to 10 subsamples drawn from a Bernoulli (0.6) distribution. Each subsample yielded a simple tree. The cutoff point was age 84.5 for five subsamples, age 85.5 for one subsample, age 82.5 for yet another subsample, severe frailty (as opposed to no frailty or moderate frailty) for one subsample and multimorbidity 3.5 for two subsamples. Therefore, although the cutoffs proposed are the most frequent in the subsamples analysed, they may not be considered optimal in general.

Thus, following this reduction process, the only variables remaining in the analysis were Physical Health as a domain of quality of life, and age (categorized), frailty status (categorized) and the number of chronic conditions (categorized) as explanatory variables. Then, a three-way ANOVA was conducted. Since no statistically significant interaction effects were observed, a fairly simple additive model was chosen. Residual diagnostics indicated that the model assumptions were met, including the normality of the residuals (Shapiro–Wilk test: *p* = 0.485). As a result, the final model yielded F(4,67) = 9.599, *p* < 0.001, and η^2^ = 0.364. In the partial contrasts, statistically significant effects were observed for age (*p*< 0.001), frailty status (*p* = 0.020), and number of chronic conditions (*p* = 0.012), all treated as categorical variables. Therefore, the three explanatory variables were retained in the model. Table 3 presents the differences in the Physical Health QoL domain between groups in terms of estimated marginal means ± SE (standard error) and effect sizes (η^2^), according to age, frailty status, and number of chronic conditions.

**Figure 1:**
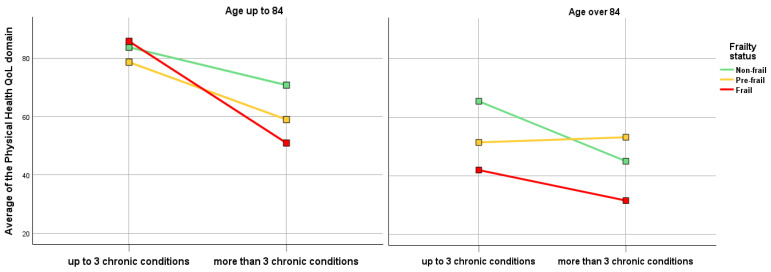
Average of the Physical Health QoL domain according to age, number of chronic conditions, and frailty status.

Table 3 presents additive model-based estimated marginal means rather than the raw group averages. According to this, participants aged >84 years exhibited, on average, an 18.86-point reduction in the Physical Health QoL domain score compared with younger participants. Similarly, individuals with more than three chronic conditions showed a mean decrease of 12.56 points compared with those with three or fewer conditions. Regarding frailty status, participants classified as pre-frail scored, on average, 5.91 points lower than non-frail individuals, whereas frail participants exhibited a mean reduction of 18.29 points compared with the non-frail group, but Bonferroni post hoc comparisons indicated that the only statistically significant pairwise difference was observed between frail and non-frail participants (*p* = 0.016). Given the additive structure of the model, the estimated means derived from this approach correspond closely to the values observed across the 12 possible subgroup combinations illustrated in Figure 1.

**Figure 2:**
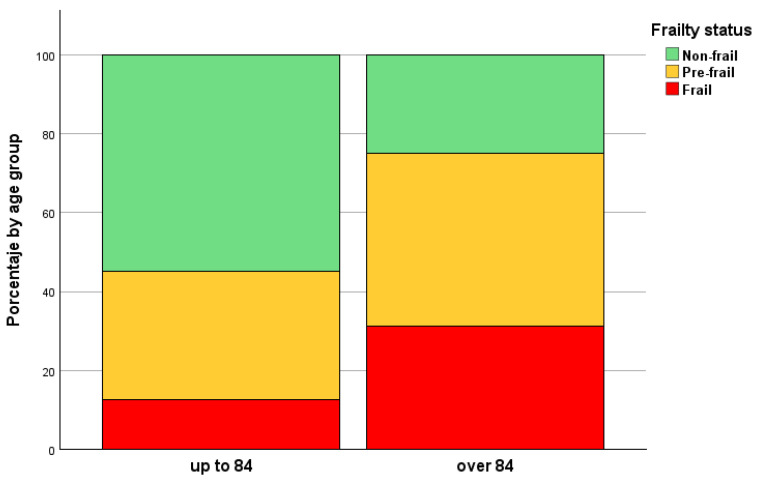
Distribution of frailty status according to age group.

The distributions of categorized age, number of chronic conditions, and frailty status is also presented in Table 1. According to the χ^2^ test, no significant association was observed between number of chronic conditions and age (*p* = 0.366), nor between number of chronic conditions and frailty status (*p* = 0.948). However, as expected, a statistically significant association was found between age and frailty status (*p* = 0.024), with frailty and pre-frailty being more prevalent among participants aged > 84 years. This distribution is illustrated in Figure 2.

## 4. Discussion

The present study examined the combined influence of age, multimorbidity, and frailty on QoL in institutionalised older adults. Our findings indicate that chronological age, frailty status, and multimorbidity burden independently and additively contribute to lower scores in the Physical Health domain of the WHOQOL-BREF. More importantly, the data support a hierarchical vulnerability architecture in which chronological age appears to operate as a structural threshold, frailty may reflect systemic reserve depletion, and multimorbidity contributes to cumulative disease burden in this sample. Together, these findings are consistent with a layered model of physical QoL impairment in long-term care settings.

### 4.1. Structural Organization of Physical QoL

Using complementary statistical approaches—including correlation analysis, canonical correlation, regression tree modelling, and additive ANOVA—we identified a structured vulnerability gradient explaining 36.4% of the variance in Physical Health scores (η^2^ = 0.364; R = 0.603). In geriatric populations, where outcomes are intrinsically multifactorial and shaped by environmental and psychosocial determinants, this magnitude suggests a clinically meaningful explanatory model [28].

Longitudinal person-centred analyses further support the heterogeneity of aging trajectories. Min et al. [29] demonstrated that transitions from young-old to old-old stages are accompanied by shifts in latent symptom classes, underscoring that aging is characterized by dynamic reclassification rather than linear decline. Our findings extend this conceptual framework to physical QoL within institutionalised settings, suggesting that the vulnerability gradient reorganizes across advancing age strata.

The magnitude of the observed differences was substantial. Participants aged >84 years experienced a mean reduction of 18.86 points compared with younger residents; individuals with more than three chronic conditions showed a 12.56-point reduction; and frail individuals demonstrated an 18.29-point decrease compared with non-frail counterparts. These differences exceed the minimal clinically important differences reported for QoL instruments, indicating clinically meaningful deterioration rather than random variation.

Importantly, canonical correlation analysis supported that the Physical Health domain carried the strongest loading relative to the broader geriatric assessment profile, supporting its interpretation as a clinically meaningful proxy of functional health within institutionalised populations.

### 4.2. Age as a Structural Vulnerability Threshold

Age was identified as the main factor o vulnerability. In this sample, age > 84 years was considered as the first splitting variable, suggesting that transition into the oldest-old stage marks a structural reorganization of vulnerability. This threshold emerged empirically from a regression tree and should be interpreted cautiously, as it may be sensitive to sample structure and model specification.

Biomechanical evidence may help contextualize the functional relevance of age stratification. Chung et al. (2023) [30] demonstrated significant differences between young-old (65–74 years) and old-old (75–84 years) adults in gait speed, stride length, knee flexion angle, ankle plantarflexion, and hip joint kinetic parameters. These biomechanical alterations may reflect declining mobility and locomotor efficiency, which are closely related to physical functioning and may, in turn, influence perceived QoL in older populations. Consistent with this broader framework, the old-old group also exhibited lower EuroQol 5-Dimension scores and higher depressive symptoms, reinforcing that advancing age reflects a multidimensional reorganization of vulnerability rather than mere chronological progression, even when assessed using different quality-of-life instruments.

Similarly, longitudinal latent transition analyses have shown systematic symptom profile shifts when individuals move from young-old to old-old stages [29]. Although focused on mental health domains, these findings converge with our results in supporting the concept of age-related structural reclassification of vulnerability profiles.

Chronological age itself does not directly cause decline; rather, it indexes cumulative exposure to physiological stress, multimorbidity, and environmental challenges, functioning as a proxy of systemic vulnerability rather than an independent causal driver. From a conceptual perspective, age may reflect cumulative vulnerability associated with declining intrinsic capacity. This interpretation aligns with evidence documenting strong interrelationships between multimorbidity, frailty, and aging [31,32]. Synergistic interactions between multimorbidity and frailty are well established [33,34,35], and meta-analytic evidence supports high frailty prevalence among individuals with multimorbidity [3]. Yao et al. (2025) [9] identified age as an independent predictor of multidimensional frailty (OR = 1.19), while Anagnostou et al. (2025) [36] reported age as the most sensitive predictor of hospitalization or death (~70%).

Importantly, longitudinal analyses indicate that the independent contribution of multimorbidity to frailty transitions may attenuate in the oldest-old. Luo et al. (2023) [31] observed stronger associations between multimorbidity and frailty progression among those aged 65–74 than among individuals in the older segment of the sample. This pattern suggests that at advanced biological ages, systemic reserve depletion may overshadow the incremental impact of disease accumulation. Consistent with this framework, frailty in our cohort superseded multimorbidity as the dominant associated factor of QoL beyond the older participants in the sample.

### 4.3. Frailty as Expression of Systemic Reserve Depletion

Within the older segment of the sample, frailty emerged as the principal explanatory factor of Physical Health QoL. Frail individuals exhibited the lowest mean scores (~36 points), representing a phenotype of advanced systemic vulnerability.

Frailty, conceptualized under the Fried phenotype and extended within multidimensional frameworks [9,18], reflects multisystem dysregulation characterized by diminished physiological reserve and reduced stress resilience. In advanced age, frailty may capture global reserve depletion more comprehensively than disease count alone.

Meta-analytic evidence supports this interpretation. Kojima et al. (2016) [34] demonstrated a clear dose–response association between frailty status and QoL, with frail individuals showing pooled reductions of −12.7 points in physical components compared with robust individuals. The magnitude of decline in our institutionalised cohort exceeded these estimates, suggesting amplification of frailty-related vulnerability within long-term care environments. These findings are consistent with observational evidence indicating that frailty is strongly associated with lower health-related QoL across multiple domains, including physical functioning, mental health, and daily activity limitations [11].

Mechanistically, frailty integrates sarcopenia, chronic inflammation, gait and balance impairment, nutritional vulnerability, sleep disturbance, depressive symptoms, and fear of falling. Yao et al. (2025), Montero-Odasso et al. (2022), and Dent et al. (2023) [9,17,18] highlight these interconnected pathways. Biomechanical data further support this interpretation: Chung et al. (2023) [30] documented increased knee flexion, reduced ankle plantarflexion, and diminished hip flexion moment in old-old adults—alterations reflecting compromised propulsive capacity and compensatory loading patterns likely to influence perceived physical functioning.

Longitudinal evidence from the Newcastle 85+ Study indicates that frailty transitions after age 85 are predominantly progressive, with minimal recovery and no transitions from frail to robust states [35]. Moreover, four or more chronic diseases significantly increased progression risk. These findings reinforce frailty as a consolidation of accumulated vulnerability.

Importantly, qualitative multimorbidity patterns further modify risk. Nguyen et al. (2019) [32] demonstrated that neuropsychiatric disease constellations conferred mortality risk comparable to higher frailty states, underscoring that vulnerability heterogeneity extends beyond simple disease counts.

### 4.4. Multimorbidity in the Younger Segment of Advanced Age

Among the younger segment of the sample, multimorbidity (>3 chronic conditions) emerged as the most relevant associated variable of Physical Health QoL, supporting the cumulative burden model described by Marengoni et al. [37].

Tran et al. (2022), and Wilk et al. (2024) [12,13] further demonstrate longitudinal links between multimorbidity patterns and QoL decline. Luo et al. (2023) [31] confirmed that multimorbidity accelerates transitions toward frailty while reducing recovery likelihood. Recent evidence also highlights the close relationship between multimorbidity and frailty development. Che et al. (2025) [4] reported that the number of comorbidities was significantly associated with frailty among older adults with multimorbidity, reinforcing the role of cumulative disease burden in the emergence of vulnerability in later life. Thus, in earlier stages of advanced age, disease accumulation appears to function as the primary vulnerability driver.

However, frailty stratified outcomes within this subgroup, reinforcing synergistic interactions between multimorbidity and frailty [3,34,35]. Conceptually, multimorbidity may initiate vulnerability accumulation, whereas frailty represents its systemic consolidation.

### 4.5. Institutionalization as a Distinct Vulnerability Context

A central contribution of this study lies in its exclusive focus on institutionalised older adults. While Chung et al. (2023) [30] evaluated community-dwelling populations, our findings extend the age-stratified vulnerability model to institutionalised settings, where environmental constraints and higher baseline frailty may amplify the impact of chronological age.

Lorber et al. (2023) [8] reported markedly higher frailty prevalence in institutional settings. Reduced mobility, social isolation, and care dependency may accelerate reserve depletion, suggesting that the layered vulnerability pattern differs both quantitatively and qualitatively from community-based populations. In this context, institutionalisation may act as a contextual amplifier of vulnerability, reinforcing the combined effects of age, frailty, and multimorbidity on QoL.

### 4.6. Clinical Implications

Age thresholds and multimorbidity burden, combined with frailty stratification, provide an intuitive vulnerability framework for long-term care. The regression tree may provide a conceptual framework for future validation studies that may complement comprehensive geriatric assessment. Contemporary geriatric care models increasingly emphasize patient-centred indicators, such as self-care capacity and perceived QoL, which are recognized as interrelated dimensions of comprehensive patient-centred assessment [38].

Anagnostou et al. (2025) [36] demonstrated moderate sensitivity of QoL measures for identifying high-risk individuals. Our findings reinforce QoL as both outcome and vulnerability marker. Integrating age stratification, multimorbidity burden, frailty assessment, and patient-reported QoL may enhance early detection and individualized care planning.

### 4.7. Limitations

Given the cross-sectional design, the analyses should be interpreted as exploratory and associative rather than causal or temporal. Identified thresholds may be sample-specific and should therefore be interpreted with caution. In particular, results derived from regression tree analysis in a sample of this size should be considered exploratory. The cutoff points identified in this sample should not be interpreted as definitive clinical thresholds and may vary across different populations or datasets. Therefore, they should not be considered as an inferential result and would benefit from validation in independent samples.

The relatively moderate sample size may limit statistical power and the ability to detect interaction effects, while also increasing the potential for model instability, particularly in the context of multiple analytical approaches. Nevertheless, the variable reduction strategy applied may have contributed to improving model robustness.

The categorization of continuous variables, although enhancing interpretability, may reduce statistical precision and obscure potential dose–response relationships. In addition, the use of convenience sampling and the exclusion of participants with severe cognitive impairment or significant functional limitations may have resulted in a sample representing a relatively more assessable subgroup, which could limit generalizability to the broader institutionalized population.

Finally, biological markers were not incorporated, and residual confounding cannot be excluded.

### 4.8. Implications for Future Research

Longitudinal studies should validate threshold stability and clarify causal pathways linking multimorbidity accumulation, frailty progression, and QoL decline. Evidence from the Newcastle 85+ cohort highlights predominantly progressive frailty transitions after age 85 [35], underscoring the need for early intervention.

Clarifying directionality between frailty and QoL deterioration remains essential [34]. Mechanistic research integrating inflammatory biomarkers, sarcopenia indicators, sleep disturbances, and depressive symptoms may refine vulnerability cascade models. Detailed biomechanical profiling, as applied by Chung et al. (2023) [30], may further elucidate physical QoL impairment mechanisms in institutionalised oldest-old adults. Further research should refine and validate risk stratification models specifically tailored to long-term care environments.

## 5. Conclusions

In this sample of institutionalised older adults, higher age, greater frailty, and a higher number of chronic conditions were associated with lower physical health-related quality of life, with the WHOQOL-BREF Physical Health domain showing the strongest relationship with the geriatric variables assessed.

The analyses suggest the presence of age-related differences in the pattern of association between frailty, multimorbidity, and physical quality of life. In this context, the older segment of the sample emerged in the regression tree as an exploratory splitting point associated with lower Physical Health scores. Within this sample, frailty showed a stronger association with physical QoL in older participants, whereas multimorbidity appeared more relevant in younger segments of the sample; however, these findings should be interpreted cautiously, as they may be sample-dependent and do not imply stable thresholds or hierarchical structures.

These results should be considered exploratory and context-dependent, given the cross-sectional design, sample size, and analytical approach. No conclusions can be drawn regarding directionality or causal relationships.

## Figures and Tables

**Table 1:** Descriptive characteristics of the study sample (*N* = 72).

		Mean ± SD/Median (IQR)	*n* (%)
Gender	Male		18 (25)
	Female		54 (75)
Age		80.65 ± 9.75	
Age (categorized)	up to 84		40 (55.6)
	over 84		32 (44.4)
Number of chronic conditions		4.71 ± 2.18	
Number of chronic conditions (categorized)	up to 3		23 (31.9)
	more than 3		49 (68.1)
Number of previous falls		0.35 ± 0.65/0 (1)	
Previous wounds	None		62 (86.1)
	One		8 (11.1)
	Two		2 (2.8)
Frailty		1.28 ± 1.36/1 (2)	
Frailty (categorized)	Non-frail		30 (41.7)
	Pre-frail		27 (37.5)
	Frail		15 (20.8)
Fall risk		4.35 ± 1.44	
Fall risk (categorized)	Low fall risk		1 (1.4)
	High fall risk		71 (98.6)
Balance and gait		16.71 ± 8.59	
Balance and gait (categorized)	No fall risk		18 (25)
	Low fall risk		15 (20.8)
	High fall risk		39 (54.2)
Nutritional status		22.36 ± 2.57	
Nutritional status (categorized)	Normal nutritional status		28 (38.9)
	Risk of malnutrition		41 (59.6)
	Malnutrition		3 (4.2)
Basic activities of daily living		65.42 ± 29.08	
Basic activities of daily living (categorized)	Independent		14 (19.4)
	Slight dependency		2 (2.8)
	Moderate dependency		29 (40.3)
	Severe dependency		18 (25)
	Total dependency		9 (12.5)
Instrumental activities of daily living		1.74 ± 1.70/1 (1)	
Instrumental activities of daily living (categorized)	Independent		1 (1.4)
	Slight dependency		4 (5.6)
	Moderate dependency		3 (4.2)
	Severe dependency		18 (25)
	Total dependency		46 (63.9)
WHOQOL-BREF domains			
Physical Health		59.38 ± 23.30	
Psychological Health		61.57 ± 17.79	
Social Relationships		55.44 ± 8.97	
Environment		73.65 ± 12.40	
Overall quality of life (WHOQOL-BREF general item)		62.51 ± 13.54	

**Table 2:** Pearson’s r correlations between geriatric assessment scores and WHOQOL-BREF domains.

*N* = 72	Age	(1)	(2)	(3)	(4)	(5)	(6)	(7)	(8)	(9)
(1) Frailty	0.351 **									
(2) Fall risk	0.378 **	0.348 **								
(3) Nutritional status	−0.122	−0.481 ***	−0.198							
(4) Instrumental activities of daily living	−0.236 *	−0.311 **	−0.095	0.465 ***						
(5) Basic activities of daily living (BADL)	−0.375 **	−0.691 ***	−0.410 ***	0.419 ***	0.424 ***					
(6) Balance and gait (Tinetti)	−0.306 **	−0.689 ***	−0.428 ***	0.319 **	0.358 **	0.842 ***				
(7) Physical Health QoL domain	−0.442 ***	−0.456 ***	−0.401 ***	0.241 *	0.154	0.388 ***	0.379 **			
(8) Psychological Health QoL domain	−0.266 *	−0.264 *	−0.265 *	0.316 **	0.173	0.190	0.180	0.778 ***		
(9) Social Relationships QoL domain	−0.242 *	−0.329 **	−0.076	0.280 *	0.235 *	0.286 *	0.260 *	0.513 ***	0.516 ***	
(10) Environment QoL domain	−0.265 *	−0.239 *	−0.260 *	0.320 **	0.180	0.202	0.158	0.622 ***	0.771 ***	0.532 ***

Notes. Colors indicate the direction and strength of the correlations: the yellow-to-orange scale represents negative correlations, and the light-to-dark green scale represents positive correlations. Bold values indicate statistically significant results after Bonferroni correction. * *p* < 0.05; ** *p* < 0.001; *** *p* < 0.001.

**Table 3:** Estimated marginal means (± SE) for the Physical Health QoL domain according to age, frailty status, and number of chronic conditions.

		Physical Health QoL Domain (Mean ± SE)	Effect Size, ANOVA
Age	up to 84 (*n* = 40)	67.98 ± 3.50	η^2^ = 0.186, *p* < 0.001
	over 84 (*n* = 32)	49.12 ± 3.46	
Number of chronic conditions	up to 3 (*n* = 23)	64.83 ± 4.06	η^2^ = 0.090, *p* = 0.012
	over 3 (*n* = 49)	52.27 ± 2.83	
Frailty status	Non-frail (*n* = 30)	66.62 ^a^ ± 3.73	η^2^ = 0.111, *p* = 0.020
	Pre-frail (*n* = 27)	60.71 ^a,b^ ± 3.81	
	Frail (*n* = 15)	48.33 ^b^ ± 5.08	

^a,b^ Groups not sharing the same superscript differ significantly according to Bonferroni post hoc comparisons.

## Data Availability

The data presented in this study are available upon request from the corresponding author due to ethical reasons.

## Supplementary Materials

The following supporting information can be downloaded at: https://www.mdpi.com/article/10.3390/healthcare14111491/s1, Figure S1: Regression tree model for the Physical Health QoL domain.

## Abbreviations

The following abbreviations are used in this manuscript:

QoL	Quality of Life
MNA	Mini Nutritional Assessment
STROBE	Strengthening the Reporting of Observational Studies in Epidemiology
IADL	Instrumental Activities of Daily Living
ADL	Basic Activities of Daily Living
OLS	Ordinary Least Squares
