# Supplementary material for: Patterns of Vulnerability: Frailty, Multimorbidity, and Physical Health-Related Quality of Life in Institutionalised Older Adults
Source: Healthcare (Basel). 2026 May 27;14(11):1491. doi: 10.3390/healthcare14111491 (PMC13257142; doi:10.3390/healthcare14111491)
Supplement: Supplementary file 1 [file healthcare-14-01491-s001.zip › healthcare-4263982-supplementary.pdf]

healthcare

MDPI

Supplementary Material

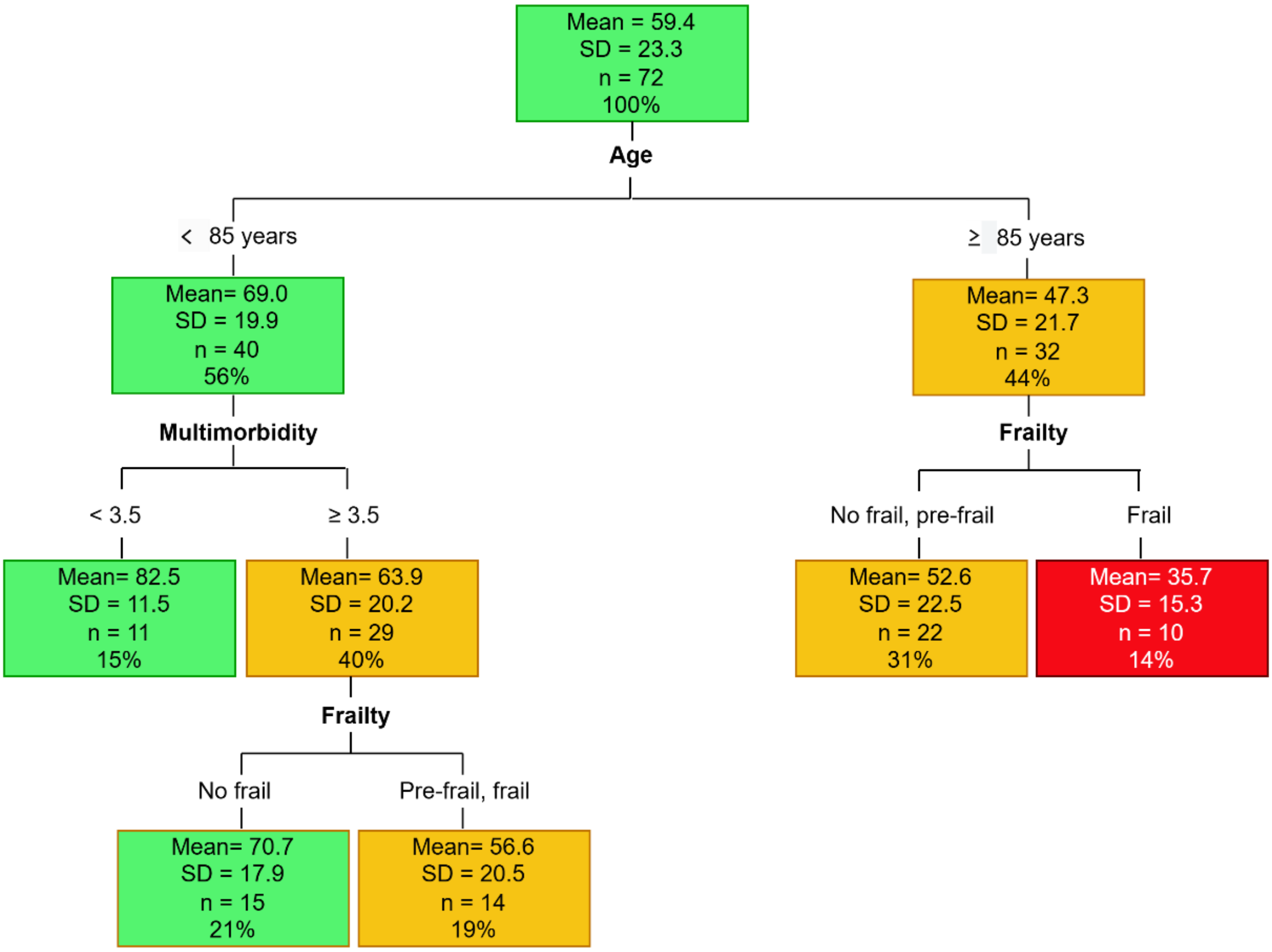

Supplementary Figure S1. Regression tree model for the Physical Health QoL domain.
